# Imaging Insights of Isolated Idiopathic Dystonia: Voxel-Based Morphometry and Activation Likelihood Estimation Studies

**DOI:** 10.3389/fneur.2022.823882

**Published:** 2022-04-26

**Authors:** Yunhao Wu, Chao Zhang, Yufei Li, Jie Feng, Ming Zhang, Hongxia Li, Tao Wang, Yingying Zhang, Zhijia Jin, Chencheng Zhang, Yuyao Zhang, Dianyou Li, Yiwen Wu, Hongjiang Wei, Bomin Sun

**Affiliations:** ^1^Department of Neurosurgery, Center for Functional Neurosurgery, Ruijin Hospital, Shanghai Jiao Tong University School of Medicine, Shanghai, China; ^2^School of Biomedical Engineering, Shanghai Jiao Tong University, Shanghai, China; ^3^Department of Neurology and Institute of Neurology, Rui Jin Hospital, Shanghai Jiao Tong University School of Medicine, Shanghai, China; ^4^Department of Radiology, Rui Jin Hospital, Shanghai Jiao Tong University School of Medicine, Shanghai, China; ^5^School of Information Science and Technology, ShanghaiTech University, Shanghai, China

**Keywords:** isolated idiopathic dystonia, voxel-based morphometry, activation likelihood estimation, grey matter volume, deep brain stimulation

## Abstract

The understanding of brain structural abnormalities across different clinical forms of dystonia and their contribution to clinical characteristics remains unclear. The objective of this study is to investigate shared and specific gray matter volume (GMV) abnormalities in various forms of isolated idiopathic dystonia. We collected imaging data from 73 isolated idiopathic dystonia patients and matched them with healthy controls to explore the GMV alterations in patients and their correlations with clinical characteristics using the voxel-based morphometry (VBM) technique. In addition, we conducted an activation likelihood estimation (ALE) meta-analysis of previous VBM studies. Our study demonstrated widespread morphometry alterations in patients with idiopathic dystonia. Multiple systems were affected, which mainly included basal ganglia, sensorimotor, executive control, and visual networks. As the result of the ALE meta-analysis, a convergent cluster with increased GMV was found in the left globus pallidus. In subgroup VBM analyses, decreased putamen GMV was observed in all clinic forms, while the increased GMV was observed in parahippocampal, lingual, and temporal gyrus. GD demonstrated the most extensive GMV abnormalities in cortical regions, and the aberrant GMV of the posterior cerebellar lobe was prominent in CD. Moreover, trends of increased GMV regions of the left precuneus and right superior frontal gyrus were demonstrated in the moderate-outcome group compared with the superior-outcome group. Results of our study indicated shared pathophysiology of the disease-centered on the dysfunction of the basal ganglia-thalamo-cortical circuit, impairing sensorimotor integration, high-level motor execution, and cognition of patients. Dysfunction of the cerebello-thalamo-cortical circuit could also be involved in CD especially. Finally, the frontal-parietal pathway may act as a potential marker for predicting treatment outcomes such as deep brain stimulation.

## Introduction

Dystonia is a rare hyperkinetic movement disorder characterized by sustained or intermittent muscle contractions causing abnormal, repetitive movements, postures, or both (1). Dystonia is often markedly disabling, and the prevalence is estimated at 16 per 100,000 patients (2). According to the high heterogeneity of the disease, the current diagnosis is based on a new classification system with two axes separately focusing on clinical characteristics and etiology (1). Isolated idiopathic dystonia, occupying major forms of the disease, refers to syndromes in which dystonia is the only motor symptom without pathological changes in the brain or inherited factors. The specific mechanisms underlying the progression of isolated idiopathic dystonia are still not fully understood.

Dysfunction of the basal ganglia resulting in the loss of inhibition of motor programs has been traditionally considered the major determinant in the pathophysiology of isolated idiopathic dystonia. Studies on early acquired dystonia have demonstrated that focal lesions are mostly found in the basal ganglia (3, 4). In animal studies, selective lesions of the striatum inducing the rodents' dystonic movements (5, 6) have revealed regional metabolic activity abnormalities in the basal ganglia by positron-emission tomography (PET) (7–9). In recent years, functional and structural neuroimaging techniques have largely deepened our understanding of the pathogenesis and functional neuroanatomy of dystonia, providing more evidence on the engagement of brain regions other than basal ganglia, such as the cerebellum and its' core role in the cerebello-thalamo-cortical pathway. Data from voxel-based morphometry (VBM) analysis, diffusion tensor imaging (DTI) and functional magnetic resonance imaging studies(fMRI) (10–12) have identified structural abnormalities frequently located in the premotor, sensorimotor cortices, parietal lobe, and subcortical areas (e.g., thalamus, cerebellum, and brain stem), as well as increased/reduced connectivity within these regions.

Voxel-based morphometry is a fully automated and whole-brain image analysis technique for evaluating voxel-wise differences in gray matter volumes (GMV) and it has been widely used in studying neurological diseases (13). In cases where conventional analysis methods fail, VBM allows the detection of ultrastructural brain changes in patients with isolated idiopathic dystonia. One of the advantages of VBM is that it can be used for making quantitative comparisons of individual region volumes throughout the brain without setting *a priori* “regions of interest.”

However, the brain regions detected across different VBM studies in dystonia are not always consistent, probably due to variations in the small patient population size and the high heterogeneity of the disease itself. Several reviews of VBM studies for dystonia have been performed, including one meta-analysis (14) using the activation likelihood estimation (ALE) method (15). This meta-analysis included studies of patients with primary focal dystonia vs. healthy controls (HCs). As a result, a greater GMV was observed in the caudate, postcentral gyrus (BA2, 3, 40), and primary motor cortex (BA4), while a smaller GMV was observed in the thalamus and putamen.

Till now, several questions remain unsolved. What are the common and clinic form-specific structural changes among distinct subtypes of isolated idiopathic dystonia? Can the imaging findings be used as potential markers for predicting the efficacy of neuromodulation therapies such as deep brain stimulation (DBS)?

Here, we conducted a cross-sectional VBM study, which retrospectively included 73 patients with isolated idiopathic dystonia and HCs. The patients were further divided into four subgroups based on clinical classifications: cervical dystonia (CD) (the main type of focal dystonia), Meige syndrome (the main type of segmental dystonia), multifocal dystonia (MD), and generalized dystonia (GD). GMV comparisons within different groups and correlations with clinical characteristics were performed. We also conducted a meta-analysis of VBM studies using activation likelihood estimation and calculated results of convergence across different studies.

There are three main purposes of this study: (a) To investigate the shared and overlapped GMV alterations of isolated idiopathic dystonia with different clinic forms, which may indicate the common pathway of the disease and help diagnosis. (b) To investigate the specific GMV alterations in subgroups of dystonia, which were divided according to distinct body distributions. (c) To explore whether the GMV alterations in some regions may correlate to clinical characteristics or imply different surgical outcomes such as globus pallidus internus (GPi) DBS. Results of the study will add evidence to the current literature and may help to identify the potential marker of disease diagnosis and treatment prediction.

## Methods

### VBM Analysis

#### Participants

This study design was approved by the appropriate ethics review board and informed consent was obtained from all individual participants included in the study.

A total of 73 patients with medication refractory dystonia (36 women and 37 men; mean age 43.04 ± 18.23 years) from the Ruijin Hospital, Shanghai, China, and 73 age- and sex-matched healthy volunteers with no histories of neurological diseases were included in the study (demographic data are summarized in Table 1, and individual details are available in Supplementary Table S1). All the participants were right-handed. Patients were diagnosed with idiopathic dystonia by experienced neurologists, based on the 2013 Movement Disorder Society Consensus. Subgroups of the disease cohort included focal dystonia (only CD was included in subgroup analysis), segmental dystonia (only Meige syndrome was included in subgroup analysis), MD, and GD. Exclusion criteria for both patients and HCs included: history of neurological/psychiatric disease, vascular diseases (e.g., stroke), and abnormal structural findings on magnetic resonance imaging (MRI). Patients who were suspected of hereditary dystonia were screened for genetic testing and those with confirmed genetic mutations were also excluded. Before the imaging scanning, no patient had received botulinum toxin therapy within the last three months. The mean age at disease onset and disease duration was 36.16 ± 19.56 years (range, 3–71 years) and 6.23 ± 6.78 years (range, 1 month to 30 years), respectively. All patients had been admitted to our hospital and received surgical procedures (Details of surgical options are available in Supplementary Table S1). Among them, 48 received bilateral DBS either targeting to GPi (*n* = 34) or subthalamic nucleus (STN) (*n* = 14). Disease evaluations were done at baseline and follow-up visits (the last visit was at least 1 year post-operatively), using Toronto Western Spasmodic Torticollis Rating Scale (TWSTRS) for CD and Burke–Fahn–Marsden Dystonia Rating scale (BFMDRS) for patients with other disease forms. Additionally, patients who had received GPi-DBS were divided into two groups according to their long-term clinical outcomes: the superior-outcome group (defined as ≥50% TWSTRS/BFMDRS score improvement) and the moderate-outcome group (defined as <50% TWSTRS/BFMDRS score improvement).

**Table 1:** Demographic and clinical data.

	**All patients with dystonia**	**Healthy controls**	***p*** -value ^*^
Number of subjects	73	73	/
Age (years, mean ± standard deviation)	43.04 ± 18.226	43.32 ± 17.223	0.882
Sex (Female/Male)	36/37	36/37	/
TIV (cm3)	(1.366 ± 0.135) ^*^ 10^2^	(1.437 ± 0.150) ^*^ 10^2^	0.357
Type of dystonia (number)		/	/
Focal dystonia	18		
Cervical dystonia	14		
Multifocal dystonia	14		
Segmental dystonia	28		
Meige syndrome	21		
Generalized dystonia	13		

*TIV, total intracranial volume*.

* *Comparisons for age and TIV were made between the patient group and the healthy control group using two-sample t-test*.

#### Data Acquisition and Preprocessing

All scans were acquired pre-operatively on a 3.0-T MRI scanner (GE Healthcare Signa HDx 3.0T, Piscataway, NJ, USA). T1-weighted MRI scans were acquired for each patient using a 3D magnetization-prepared rapid acquisition, gradient-echo sequence (TR = 7.0 ms, TE = 3.0 ms, flip angle = 7, slice thickness = 1.0 mm, the field-of-view = 256 × 256 mm^2^, spatial resolution = 1 × 1 × 1 mm^3^) and used as an anatomical reference for VBM analysis. An experienced radiologist (ZJ Jin) has checked all image data to assure image quality and the absence of significant brain pathology or artifacts.

#### Data Analysis

The SPM12 software running on MATLAB R2019b (MathWorks, MA) was used to identify GMV abnormalities among different patient groups. Data processing included five steps: segmentation, generation of group/subgroup average template data, spatial normalization of images to Montreal Neurological Institute (MNI) space, modulation, and smoothing. Whole-brain gray matter segmentation was performed using the unified segmentation approach, and a more accurate inter-subject alignment was further achieved using the diffeomorphic non-linear registration tool to create group average template data. Template images were registered to the SPM standard MNI space and smoothed Jacobian scaled GM images were generated using estimated deformations. Two authors (YY Zhang and M Zhang) checked the quality of segmented and normalized images independently. GM probability images were smoothed using a Gaussian filter with a 6-mm full-width half-maximum (FWHM).

#### Statistical Analysis

The general linear model based on Gaussian random field theory statistically analyzed the GMV maps. To assess voxel-wise statistical differences in GMV between whole patients and HCs, as well as in each subgroup, we performed two independent *t*-tests, including age, sex, and total intracranial volume (TIV) as nuisance variables. In group comparisons, we applied a conservative approach with a whole-brain statistical threshold of *P*_FWE_ < 0.005 at the cluster level (Bonferroni correction for multiple comparisons within five groups). Cluster (k) threshold was set at 100 voxels, referring to previous VBM studies that applied a threshold at 50–100 voxels usually.

Moreover, to evaluate the association of GMV with clinical characteristics (age of onset, disease duration, and BFMDRS scores), we performed correlation analyses using the multiple regression function in SPM12. TIV, gender, and age were included in the models as nuisance variables. Finally, independent *t*-tests between the superior-outcome group and the moderate-outcome group were also performed in patients who had received GPi-DBS, seeking to explore the potential structural GM changes associated with surgical efficacy. To avoid possible edge effects between different tissue types, absolute threshold masking was used to exclude voxels with Jacobian-modulated GM values <0.2. The significance level was set at a cluster level threshold of *P*_FWE_ < 0.05, and the trend level at uncorrected *P* < 0.001.

### Literature Search and Study Selection

We conducted a literature search on the PubMed database (www.pubmed.org) using the keywords: (“dystonia” or “Meige” or “blepharospasm” or “writer's cramp”) and (“VBM” or “voxel-based morphometry”), resulting in 57 results published from January 2011 to January 2020. The studies were considered for inclusion if they (1) reported VBM comparison between patients with isolated idiopathic dystonia and HC subjects; (2) reported whole-brain results regarding changes in a stereotactic space in three-dimensional coordinates (x, y, z); (3) used significance thresholds either corrected for multiple comparisons or uncorrected with spatial extent thresholds; and (4) were published in English after peer reviews. The studies were excluded if they met at least one of the following criteria: (1) inclusion of patients with dystonia with inherited and acquired factors; (2) reviews and meta-analyses; (3) incomplete coordinates of stereotactic space provided; and (4) no HC group was set in the study. Two authors (YH Wu and C Zhang) performed study selection independently and were confirmed by a third author (HX Li). The search strategy flow chart is described in detail in Supplementary Figure.

### Activation Likelihood Estimation

The meta-analysis was carried out using Ginger ALE software 3.0.2 (http://www.brainmap.org). GingerALE uses the analytical method of determining the null distribution of the ALE statistic. ALE was originally developed by Peter Turkeltaub (16) and can also be understood as anatomic likelihood estimation when applied in conjunction with VBM data. This method aims to reveal consistencies in various imaging studies by generating a statistical map presenting the likelihood of activation for voxels. The principle and procedure of ALE methods can be summarized as follows: All activation foci (peak voxel) were collected from different studies and their coordinates were converted to a standard space- MNI templates or Talairach. For the uncertainty of the location accuracy of the collected foci(peaks) which is limited by imaging techniques, intersubject anatomical variations, defects of algorithms, etc., activation foci are viewed as the centers of localization probability distributions. The localization probability distributions for foci were modeled by 3D Gaussian functions. Each voxel within the brain was assigned a value equal to the probability that at least one of the foci in the data set lay within the voxel, and the value is referred to as ALE. By employing the random-effects method by Eickhoff et al. (15, 17), the FWHM of the Gaussian function is derived from the subject sizes that larger subject size in a study will get a tighter Gaussian and smaller FWHM. ALE calculations first create a 3D Image for each study, which is called Modeled Activation (MA) map, and the ALE image is a union of all of the MA maps. To differentiate the random convergence (i.e., noise) from true convergence (i.e., nonrandom clustering of foci), a null distribution is computed non-parametrically by a permutation procedure. For this permutation analysis, a random gray matter voxel was selected from each MA map, and 1,000 (defined manually) sets of random coordinates within in mask volume were processed identically to the practical data. Histograms of all voxel values (ALE scores) were utilized as null hypothesis distribution. A 3D *P*-value image was created by combining the probabilities of finding each value in a MA map. By setting the *P*-value threshold, volumes above the thresholded ALE values would be defined.

Fourteen published studies (18–31) reporting 96 focal GMV abnormalities (53 increased and 43 decreased GMV data) satisfied the criteria and were enrolled in the meta-analysis, comprising a total of 387 patients with isolated idiopathic dystonia. Each literature quality was evaluated using Newcastle-Ottawa Quality Assessment Scale (NOS) (http://www.ohri.ca/programs/clinical_epidemiology/oxford.htm; Supplementary Table 2). The main characteristics of the included studies are described in Table 2. Coordinate data were extracted from each included study, and Talairach coordinates reported in one study were automatically converted to MNI space using the Lancaster transformation in Ginger ALE (32, 33). The ALE values were permuted 1,000 times to determine their significance while minimizing distributional assumptions. These maps were finally thresholded and corrected for multiple comparisons by setting the cluster level threshold at *P*_FWE_ < 0.05, calculating a minimum cluster size of 520 mm3. ALE maps were created separately for coordinates associated with increased or decreased GMV in patients compared with HCs. Mango (www.ric.uthscsa.edu/mango/) was used to visualize our ALE maps overlaid onto a high-resolution brain template, Colin27.nii, in the MNI space (www.brainmap.org/ale).

**Table 2:** Summary of inclusive studies in meta-analysis.

**Study**	**First author**	**Publication year**	**Subjects**	**Patients' number (F/M)**	**Age (mean years)**	**Handed (R/L)**	**Statistical thresholds**	**FWHM (mm)**	**Increased GMV (main clusters' number)**	**Decreased GMV (main clusters' number)**	**Clinical correlation**	**Tool**	**MRI**
1	Ramdhani et al. (18)	2014	CD&BLP vs HC	11 (7/4) +10 (9/1)	57.28 (CD); 59.2 (BLP)	R	FWE corrected, *P* <0.05	8	Y (5)	Y (5)	N	SPM8	3T
			WC&SD vs HC	11 (6/5) +12 (8/4)	52.75 (WC); 54.75 (SD)	R	FWE corrected, *P* <0.05	8	Y (12)	N	Y (disease duration and GMV)	SPM8	3T
2	Piccinin et al. (22)	2015	CCD vs HC	27 (18/9)	54.18 ± 11.70	NA	*P* <0.001 uncorrected	10	N	Y (14)	Y (age, disease duration, disease severity, BoNT and GMV)	SPM8	3T
3	Gallea et al. (26)	2018	FHD	18 (3/15)	53.94 ± 12.04	R	FWE corrected, *P* <0.001	10	N	Y (2)	N	SPM8	3T
4	Filip et al. (28)	2017	CD vs. HC	25 (15/10)	45.8 ± 12.3	R	FWE corrected, *P* <0.05	10	Y (5)	Y (2)	N	SPM8	1.5T
5	Pantano et al. (29)	2011	CD vs. HC	19 (15/4)	53.2 ± 11.2	NA	FDR corrected, *P* <0.05	12	N	Y (4)	N	SPM5	1.5T
6	Kirke et al. (30)	2017	SD & SD/DTv vs HC	40 (34/6)	54.4 ± 8.3 (SD); 60.0 ± 10.1 (SD/DTv)	NA	FWE corrected, *P* <0.05	NA	Y (4)	N	Y (onset age, voice tremor and GMV)	SPM8	3T
7	Delnooz et al. (31)	2015	CD vs HC	23 (14/9)	57.3 ± 9.8	R (21) / L (2)	FWE corrected, *P* <0.05	10	N	Y (1)	Y (disease duration, disease severity and GMV)	SPM8	3T
8	Prell et al. (20)	2013	CD vs. HC	24 (18/6)	52	NA	FWE corrected, *P* <0.05	8	Y (6)	Y (4)	Y (disease severity and GMV)	SPM2	1.5T
9	Cerasa et al. (21)	2014	DT vs. HC	12 (6/6)	62.9 ± 15	NA	FWE corrected, *P* <0.05; *P* <0.001 uncorrected	8	Y (3, 1 at FWE corrected level)	N	N	SPM8	3T
10	Simonyan et al. (27)	2012	SD vs. HC	40 (25/15)	56.9 ± 10.6	R	FWE corrected, *P* ≤ 0.01	10	Y (5)	N	Y (disease severity and GMV)	SPM8	NA
11	Granert et al. (23)	2011	MC vs. HC	11 (2/9)	43	R	*P* <0.001 uncorrected	10	Y (4)	Y (4)	Y (piano-playing skill and GMV)	SPM5	3T
12	Mantel et al. (19)	2018	WC vs. HC	26 (11/15)	46.8 ± 13.7	NA	FWE corrected, *P* <0.05;FDR corrected, *P* <0.05	8	N	Y (2)	Y (disease severity and GMV)	SPM12	3T
13	Zeuner et al. (24)	2015	WC vs. HC	22 (13/9)	50.7 ± 12.2	R	FWE corrected, *P* <0.05	12	Y (2)	N	Y (disease duration and GMV)	SPM8	3T
14	Bianchi et al. (25)	2019	TSFD vs. HC	16 (8/8)	45.3 ± 10.8	R	FWE corrected, *P* <0.01	6	Y (2)	N	N	SPM12	3T

*F, female; M, male; R, right; L, left; FWHM, full-width at half maximum; GMV, gray matter volume; CD, cervical dystonia; BLP, blepharospasm; WC, writer's cramp; HC, healthy control; SD, laryngeal dystonia; CCD, craniocervical dystonia; FHD, focal hand dystonia; DT, dystonic tremor; DTv, dystonic tremor of voice; MC, musician's cramp; TSFD, task specific focal dystonia; FWE, family wise error; FDR, false discovery rate; BoNT, botulinum toxin; Y, reported cluster(s); N, no reported cluster; NA, not available*.

## Results

### Clinical Data and Volumetric Analysis

No significant differences were found in the age and TIV between patients with dystonia and HCs [mean ± SD: age: 43.04 ± 18.226 vs. 43.32 ± 17.223 years, *P* = 0.882; TIV: (1.366 ± 0.135) ^*^ 10^2^ cm3 vs. (1.437 ± 0.150) ^*^ 10^2^ cm3, *P* = 0.357; Table 1].

#### GMV Alterations in Patients With Isolated Idiopathic Dystonia vs. Healthy Controls

**Figure 1:**
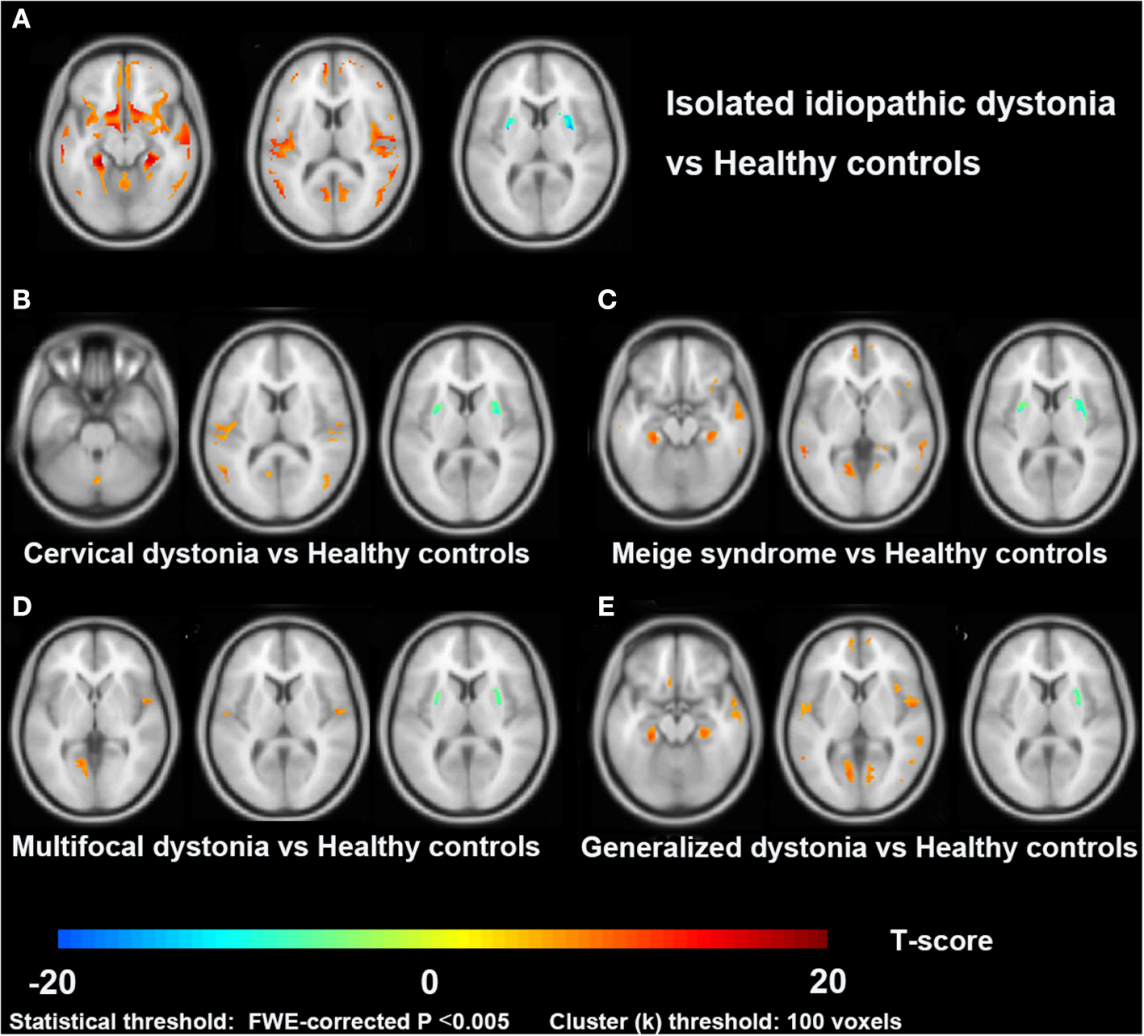
Cross-sectional comparisons between patients with dystonia and healthy controls in the VBM study. Slice view **(A–E)** of gray matter volume changes in patients with isolated idiopathic dystonia, and also in each patient subgroup (cervical dystonia, Meige syndrome, multifocal dystonia, and generalized dystonia), compared to healthy controls.

As shown in Figure 1, dystonia patients demonstrated an abnormal increase of GMV in extensive clusters encompassing the cerebral cortex, cerebellum, and basal ganglia compared to HCs (*P*_FWE_ < 0.005). The GMV in the bilateral temporal gyrus, parahippocampal gyrus, left frontal gyrus (mainly located in left BA9, 10, 44), left pre/postcentral gyrus (BA 4), and cerebellum posterior lobe (vermis_4_5, vermis_7, and vermis_8) was significantly higher in dystonia patients than in HCs. Meanwhile, a lower GMV of patients was observed in the bilateral putamen. All GMV changes between patients and HCs are described in Table 3.

**Table 3:** Gray matter volume changes in the whole group and subgroups (cervical dystonia, Meige syndrome, multifocal dystonia, and generalized dystonia) of Isolated idiopathic dystonia, compared with healthy controls (*P*_FWE_ < 0.005 at cluster level).

**Peak MNI coordinate**								
**Brain region**	**Side**	**BA**	**AAL 116**	**X**	**Y**	**Z**	**Peak intensity (T-score)**	Cluster size^*^(Voxel number)
**Isolated idiopathic dystonia vs. Health controls**								
**Gray matter volume increase**								
Parahippocampal Gyrus	R	-	ParaHippocampal_R	24	−35	−9	18.67	822
	L	-	ParaHippocampal_L	−21	−38	−9	18.75	443
Middle Temporal Gyrus	R	-	Temporal_Mid_R	62	−36	−3	14.22	1,952
	L	-	Temporal_Mid_L	−60	−39	−5	16.21	1,072
Superior frontal gyrus	L	9	Frontal_Sup_L	−23	44	38	9.10	186
Middle frontal gyrus	L	10	Frontal_Mid_L	−33	51	12	9.21	296
Inferior frontal gyrus	L	44	Precentral_L	−57	4.5	19.5	10.03	179
Precentral gyrus	L	4	Postcentral_L	−42	−15	51	10.11	142
Cerebellum posterior lobe	-	-	Vermis_8	0	−65	−29	12.07	102
	-	-	Vermis_7	5	−75	−29	7.45	104
	-	-	Vermis_4_5	0	−57	−16.5	10.99	219
Inferior temporal gyrus	L	-	Temporal_Inf_L	−48	−53	−26	10.43	131
**Gray matter volume decrease**								
Putamen	R	-	Putamen_R	29	5	9	−18.07	208
	L	-	Putamen_L	−30	−2	8	−16.39	172
**Cervical dystonia vs. Health controls**								
**Gray matter volume increase**								
Parahippocampal gyrus	R	-	ParaHippocampal_R	23	−35	−9	10.82	316
	L	-	ParaHippocampal_L	−21	−38	−9	11.08	191
Lingual gyrus	R	-	Lingual_R	12	−62	2	7.66	228
	L	-	Lingual_L	−14	−60	0	8.76	403
Middle frontal gyrus	R	6	Frontal_Sup_R	26	9	57	7.95	140
	R	6	Precentral_R	48	2	39	7.69	206
Paracentral lobule	L	5	Precuneus_L	−6	−45	56	7.13	170
Middle temporal lobe	L	21, 39	Temporal_Mid_L	−60	−38	−5	9.32	178
Inferior parietal lobule	R	-	Parietal_Inf_R	51	−48	38	6.82	153
	L	40	Parietal_Inf_L	−36	−50	50	7.55	149
Middle occipital gyrus	R	19	Occipital_Mid_R	42	−77	2	7.33	273
Postcentral gyrus	L	4	Postcentral_L	−44	−17	51	7.39	141
Cerebellum posterior lobe	-	-	Vermis_7	−2	−68	−29	7.21	101
**Gray matter volume decrease**								
Putamen	R	-	Putamen_R	30	0	8	−12.10	178
	L	-	Putamen_L	−30	−3	6	−10.69	154
**Meige syndrome vs. Health controls**								
**Gray matter volume increase**								
Parahippocampal gyrus	R	-	ParaHippocampal_R	26	−36	−8	11.77	421
	L	-	ParaHippocampal_L	−23	−36	−9	11.74	266
Lingual gyrus	R	-	Lingual_R	24	−47	−8	8.27	194
	L	-	Lingual_L	−20	−53	−5	10.07	375
Superior frontal gyrus	R	6	Frontal_Sup_R	26	5	57	8.10	127
Medial frontal gyrus	L	-	Frontal_Sup_Medial_L	−9	62	6	8.52	296
Middle temporal gyrus	R	-	Temporal_Mid_R	62	−38	−3	11.02	1,031
	L	-	Temporal_Mid_L	−59	−41	−3	11.8	665
Inferior parietal lobule	R	-	Rolandic_Oper_R	54	−29	23	8.78	350
	R	-	Parietal_Inf_R	51	−50	39	8.21	138
Precuneus	R	7	Precuneus_R	11	−69	32	8.85	210
	L	7	Precuneus_L	−8	−62	51	8.82	329
**Gray matter volume decrease**								
Putamen	R	-	Putamen_R	30	3	9	13.25	180
**Multifocal dystonia vs. health controls**7								
**Gray matter volume increase**								
Parahippocampal gyrus	R	-	ParaHippocampal_R	21	−36	−6	8.11	195
	L	-	ParaHippocampal_L	−23	−36	−11	8.33	193
Lingual gyrus	L	-	Lingual_L	−18	−51	-−5	8.47	290
Superior temporal gyrus	R	-	Rolandic_Oper_R	50	−9	9	7.50	137
**Gray matter volume decrease**								
Putamen	R	-	Putamen_R	30	0	9	−8.97	134
	L	-	Putamen_L	−29	−3	8	−8.60	126
**Generalized dystonia vs. health controls**								
**Gray matter volume increase**								
Parahippocampal gyrus	R	-	ParaHippocampal_R	26	−38	−6	10.72	333
	L	-	ParaHippocampal_L	−23	−36	11	11.80	249
Lingual gyrus	R	-	Lingual_R	23	−48	−6	9.19	192
	L	19	Lingual_L	−12	−63	2	9.67	431
Superior frontal gyrus	L	-	Frontal_Sup_Medial_L	−8	51	33	7.19	119
Middle frontal gyrus	R	6	Frontal_Sup_R	26	11	57	7.64	159
Medial frontal gyrus	R	-	Frontal_Sup_Medial_R	9	60	14	7.31	174
Inferior frontal gyrus	R	9	Precentral_R	51	5	32	7.22	212
Superior temporal gyrus	R	22	Rolandic_Oper_R	53	2	0	9.92	391
Middle temporal gyrus	R	21 39	Temporal_Mid_R	63	−27	−12	7.96	455
	L	-	Temporal_Mid_L	−60	−35	−6	9.02	386
Transverse temporal gyrus	L	41	Temporal_Sup_L	−57	−23	12	7.91	283
Precuneus	R	7	Precuneus_R	9	−72	35	8.63	105
	R	7	Cuneus_R	14	−72	24	6.42	112
Inferior parietal lobule	L	-	Parietal_Inf_L	−38	−50	48	7.59	170
	R	-	Parietal_Inf_R	41	56	47	7.85	332
**Gray matter volume decrease**								
Putamen	R	-	Putamen_R	29	0	9	9.12	130

*BA, Brodmann's Area; AAL, Anatomical Automatic Labeling; R, right; L, left; -, not defined*.

* Cluster size at brain region (Voxel: 1.5 mm ^*^1.5 mm ^*^1.5 mm in MNI space and 1 mm^*^1 mm^*^1 mm in raw data).

#### GMV Alterations in Patient Subgroups vs. Healthy Controls

Among four subgroups, the increased GMV of GD compared to HC in cortical brain regions was the most extensive, while cortical alterations in the MD group were the least. Increased GMV in the para-hippocampal gyrus, lingual gyrus, and temporal gyrus was observed in all subgroups (CD, Meige syndrome, MD, and GD). Moreover, an increased GMV was found in the frontal gyrus and inferior parietal lobule of patients with CD, Meige syndrome, and GD, and the GMV alteration of the cerebellum posterior lobe (vermis 8) especially occurred in CD.

#### Correlation With Clinical Characteristics

**Figure 2:**
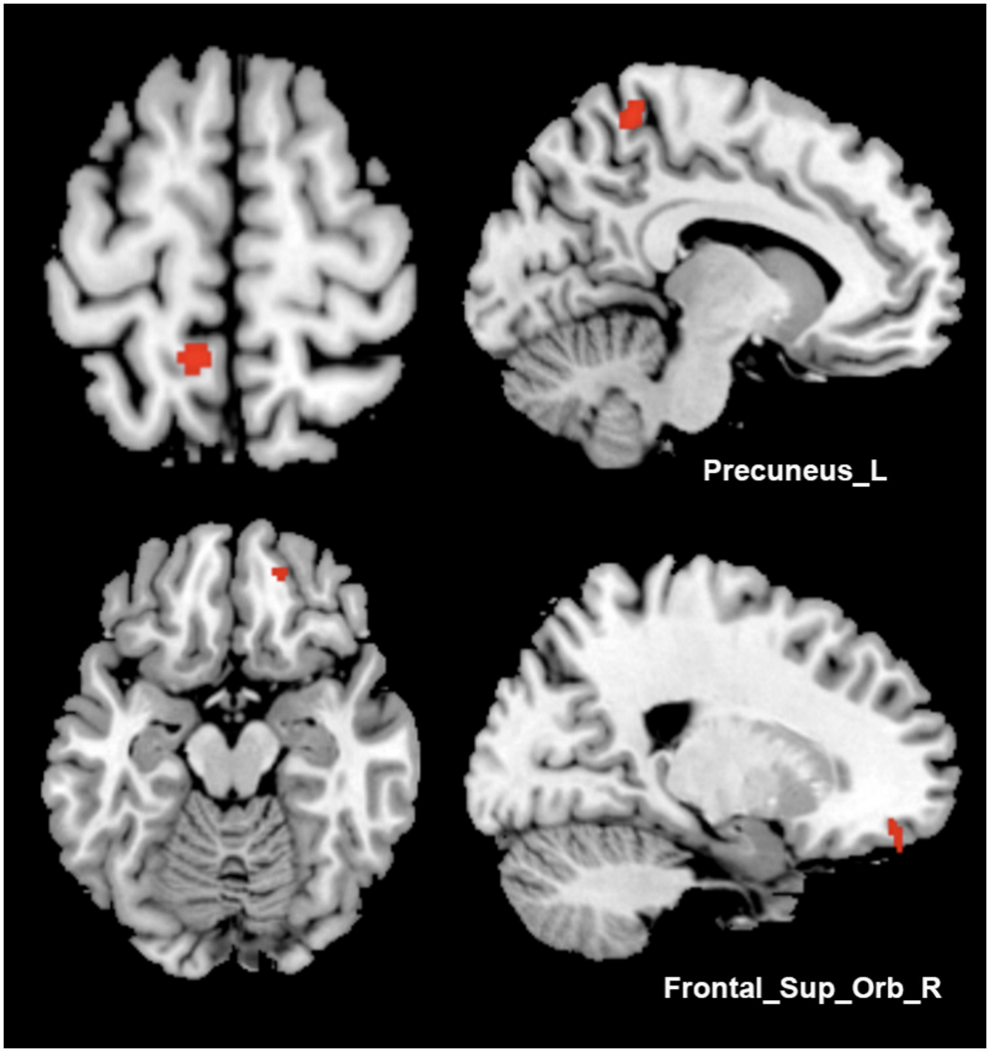
Comparisons between different surgical outcome groups. Increased gray matter volume of the moderate-outcome group was shown in Precuneus_L and Frontal_Sup_Orb_R, compared to the superior-outcome group (axial and sagittal views).

No statistically significant correlation was found between clinical characteristics, TWSTRS/BFMDRS improvement, and GMV. As a result of an independent *t*-test between two outcome groups of patients who received bilateral GPi-DBS, we found trends of increased GMV of the moderate-outcome group in the left precuneus (peak MNI coordinates: −9, −45, 64.5; peak *T*-value: 3.74; voxel number: 139; *P* < 0.001, uncorrected) and right orbitofrontal gyrus (peak MNI coordinates: 18, 48, −16.5; peak *T*-value: 3.45; voxel number: 58; *P* < 0.001, uncorrected) (Figure 2).

### Meta-Analysis Results

**Figure 3:**
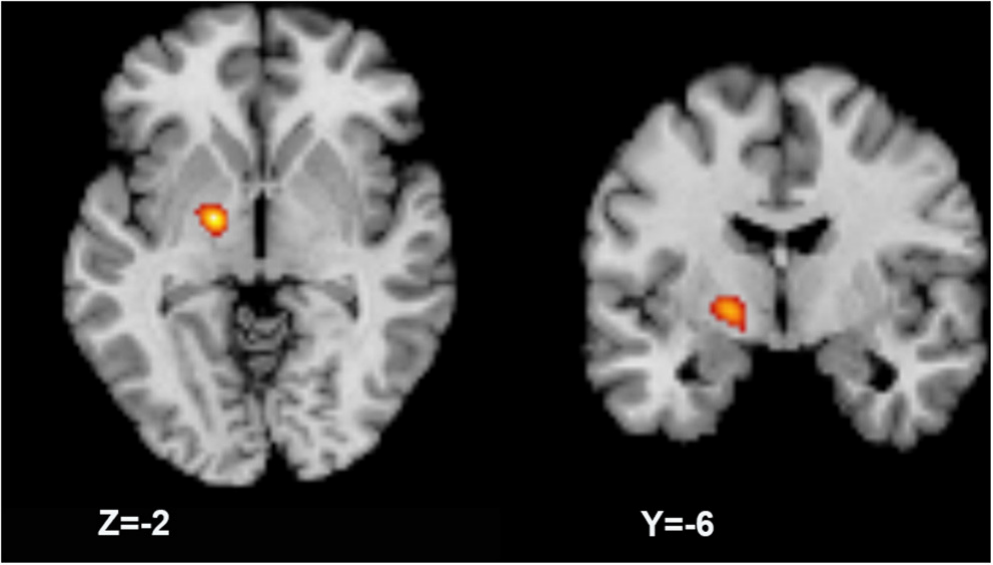
Activation likelihood estimation (ALE) meta-analysis result. The meta-analysis result shows a larger gray matter volume in the left globus pallidus (axial and coronal views).

In one cluster, increased GMV was identified in the left medial and lateral globus pallidus (peak MNI coordinates: −18, −6, −2; peak ALE value: 0.017; volume size: 816 mm3; peak *P*-value: 2.1^*^10^−7^) of patients compared with that in HCs (Figure 3); no significant decreases in GMV were identified.

## Discussion

This VBM study reviewed images of 73 patients with various forms of isolated idiopathic dystonia (focal, segmental, multifocal, and generalized dystonia), comprising one of the largest dystonia cohorts in a single-center till now. Results of our studies have revealed widespread GMV alterations involving somatosensory, motor, limbic and visual systems, supporting dystonia as a network disorder (34).

Basal ganglia have been historically regarded to play a key role in the pathophysiology of dystonia. One prevailing theory is the imbalance between the direct excitatory and indirect inhibitory output pathways, that the dysfunction of basal ganglia's inhibitory projections leads to excessive movement (35). Evidence from acquired dystonia has shown that the lesions in the putamen and caudate were the most common causes of the disease (36). Interestingly, the significant decreases of GMV in putamen were shared in four subgroups as well as the whole cohort of patients, when compared to HCs. In addition, the result is consistent with the findings of previous studies including the meta-analysis (14), which highly indicates the causal role of putamen's atrophy in dystonia. While early studies mainly focused on focal dystonia, we expanded the subject scale and performed analysis including other clinical forms to improve the reliability of the results. Moreover, the meta-analysis has identified increased GMV in the left globus pallidus, in accordance with increased GMV and activity of globus pallidus frequently reported in focal dystonia. In an anatomic aspect of basal ganglia, the medial and lateral portions of globus pallidus (GPi and globus pallidus externus) received inhibitory projections from putamen, thus decreased inhibitory signals derived from putamen would lead to overactivity of globus pallidus externus (GPe). We suppose these may indicate compensatory alterations of pallidum located within the basal ganglia network. Nevertheless, the meta-analysis result was found in a very limited region. Convergent clusters' localizations defined by ALE are limited and easily altered by the varieties of sample sizes, imaging techniques used, smoothing kernels applied, and significance levels set in different studies, as the information of studies concluded in Table 2. The scattered aberrances in cortical regions would tend to be eliminated during ALE calculation, leaving the significant clusters with the maximum likelihood. Also, the bias of clinical forms of the disease could be a large-weighted influencer on the current results, since focal (e.g., cervical dystonia) and task-specific forms are mainly included in the selected literatures.

In cortical regions, the GMV abnormalities of idiopathic dystonia were mainly located in the bilateral temporal gyrus, left frontal gyrus, and pre/postcentral gyrus, which were in accordance with the evidence accommodated by neurophysiology and neuropathology studies implicating cortical abnormalities in dystonia (11). The components involved somatosensory, executive control networks, and some portions of default mode, visual, and auditory networks. Since patients with dystonia can demonstrate sensory or perceptual dysfunction, under the theme of ‘defects in sensorimotor integration' from psychophysical studies (37), GMV increase in those areas may explain the sensorimotor processing deficits including dysfunction in the integration of sensory and motor information, temporal, and spatial discrimination, and even in social and language processing (38, 39). Increased GMV of bilateral parahippocampal, lingual, and temporal gyrus were also demonstrated in the whole cohort, and also four subgroups. These regions mainly participate in high-level motor execution and planning (40), auditory processing, memory encoding as well as multi-sensory integration (41, 42), supporting the interaction of multiple systems in different forms of dystonia. In subgroup analyses, abnormalities of frontal gyrus and inferior parietal lobule occurred in CD, Meige syndrome, and GD groups additionally. The frontal-parietal pathway is responsible for executive control processing procedures, of which decreased activity and functional connectivity have been demonstrated in dystonia (19, 43). These results point to the impairment of executive control shared in the above three subgroups, and the structural changes may be secondary to basal ganglia and sensorimotor networks for their increased functional connections linking to the frontal-parietal network (19). In terms of ranges of influence in cortical regions, GD presented the most extensive GMV abnormalities, especially in sensorimotor areas, probably attribute to the widely affected body regions of patients.

Significant increased GMV of the posterior cerebellar lobe (vermis IV-V, VII-VIII) was also observed exclusively in CD and the entire patient cohort. Cerebellum involvement in dystonia waas supported by genetic and pharmacologically induced animals models as well as cases that benefit from surgical interventions in the cerebellum (44). Posterior cerebellar lobe projects to sensorimotor areas through the ventrolateral thalamic nuclei, which facilitates intracortical inhibition (45, 46). Consistently, structural changes and decreased activation in posterior cerebellar lobules, and its connectivity with the prefrontal area was previously reported in CD (28), indicating the disrupted cerebello-thalamo-cortical circuit underlies the pathophysiology of dystonia, and such impairment could be especially prominent in CD. Moreover, miscommunication has been found between the cerebellar circuit and basal ganglia-thalamo-cortical circuit (28, 47).

Among the current literature on imaging studies, inconsistent results would be one of the existing issues. For instance, the thalamus is suggested to mediate focal dystonia by functional MRI studies and thalamic lesions studies (48–50), receiving inhibitory projections from the basal ganglia in the cortico-thalamic motor control loops. While thalamic volume reductions were reported in some forms of idiopathic dystonia (51), our study revealed no significant differences in the thalamus between patients and HCs. In other identical cortical or cerebellar regions, GMV could also be found to both increase and decrease in different studies. On one hand, various clinic forms and analysis methods applied in different studies may explain some of the reasons for inconsistency. Methodological limitations of VBM would prevent some subtle structural changes in the small nucleus from being detected. On the other hand, as dystonia is a network disorder, the neuron loss and compensatory formation of new synaptic connections may influence how the specific structure changes during the dynamic remodeling of the network organization (52). However, whether the morphometric changes are primary or secondary derived from other brain regions are unable to be identified currently, but future longitudinal studies with a larger sample size are necessary.

Another notable point is the asymmetric deficits in the basal ganglia and cortex concluded from VBM and meta-analysis studies. We observed that the defective cortical and subcortical structures were mostly left-lateralized, consistent with a majority of previous literature. Some have suggested that this phenomenon may be explained by asymmetric symptoms (14). Since all patients included are right-handed, it's interesting to further explore whether cerebral dominance possesses the correlation to asymmetric symptoms or lateralized GMV alterations.

Clinical correlation, however, failed to be found with GMV in any brain regions (*P*_FWE_ < 0.05). In other VBM studies of dystonia, correlations were occasionally found between GMV and disease severity or duration, however, with results mostly sporadic or significant under a loose threshold (uncorrected *P* < 0.001). These probably indicate none of the solitary strong correlations of morphometry measures with clinical characteristics exist in such network disorder, as multiple and complicated factors would affect scattered and interactive brain regions. Nevertheless, trends of increased GMV regions of the left precuneus and right superior frontal gyrus were demonstrated in a moderate-outcome group compared to the superior-outcome group in our study. GPi–DBS proves to be an effective and well-established treatment for refractory dystonia (53), but the surgical outcomes are highly variable, highlighting the importance of seeking appropriate predictors for DBS benefits (54, 55). Since increased GMV of the precuneus and superior frontal gyrus, which are mainly responsible for motor control, has been previously proved in idiopathic dystonia (both in the whole cohort and most subgroups compared to HC), we suppose the degree of GMV increase within frontal-parietal pathways may further indicate the severity of network dysfunction and take advantages in implying treatment outcomes. The severe structural impairments in those areas would lead to more difficulties in repressing dystonic symptoms. Also, previous studies have, respectively investigated the associations of the clinical outcomes with cortical integrity (56) and functional activities (57), of which results involved regions within sensorimotor related networks. Finally, multimodal imaging studies apart from VBM are also required to assist in describing comprehensive disease characteristics and their associations with clinical outcomes, which aims to screen patients likely to benefit from DBS or other invasive procedures.

This study had the following limitations: First, the number of participants was still insufficient, considering various clinic forms of dystonia. Longitudinal VBM studies are also required for tracing GMV dynamic changes with disease progression. Second, with time and labor limitations, data collection of the meta-analysis was only based on the PubMed database from the past ten years, which would likely lead to selection bias. VBM technique also has its defects, as it measures tissue density within a given voxel and extrapolates the local volume measure from the Jacobian warp matrix. Some authors have pointed out that VBM cannot replace classical volumetric analysis (e.g., manual volumetry) for its' well-defined anatomical structures, that is considered the “gold standard” (58, 59). Structural assessments of the basal ganglia and thalamus may be particularly difficult and inaccurate for automated segmentation in T1-weighted imaging (60), due to the influence of high iron content. Moreover, VBM could not directly reflect the underlying neuronal structural changes. Thus, the application of multimodal imaging combined with electrophysiological studies is essential to further solidify the pathophysiology of dystonia.

## Conclusion

In conclusion, our study included a cohort of patients with various clinical forms of idiopathic dystonia to explore their GMV abnormalities. We did an ALE meta-analysis to investigate the convergent aberrant GMV clusters based on previous VBM studies. This study demonstrated widespread morphometry alterations in patients with idiopathic dystonia. Network abnormalities of the putamen and cortical regions relating to the somatosensory and executive control networks were shared in all subgroups, indicating the common pathophysiology of the disease centered on the dysfunction of the basal ganglia-thalamo-cortical circuit. Moreover, GD demonstrated the most extensive GMV abnormalities in cortical regions, and CD featured the prominent GMV increase in the posterior cerebellar lobe, implying additional involvement in the cerebello-thalamo-cortical circuit. Finally, though no significant clinical correlation was observed in any brain regions, trends of GMV differences in the precuneus and frontal gyrus in two different GPi–DBS outcome groups point to the potential role of the frontal-parietal pathway in further predicting treatment outcomes.

## Data Availability Statement

The raw data supporting the conclusions of this article will be made available by the authors, without undue reservation.

## Ethics Statement

The studies involving human participants were reviewed and approved by Ruijin Hospital Ethics Committee Shanghai Jiao Tong University School of Medicine. The patients/participants provided their written informed consent to participate in this study.

## Author Contributions

CheZ, HW, BS, and YiW conceived and designed the study. TW, YiZ, and ZJ collected imaging data. YuW, ChaZ, JF, YL, and MZ performed data processing and statistical analysis. HL evaluated patients. DL performed surgeries. YuW and ChaZ wrote the article. CheZ, YiW, and HW reviewed and edited the manuscript. All the authors read and approved the manuscript.

## Funding

This study was supported by the National Natural Science Foundation of China (Grant number 81870887 and 61901256), Shanghai Collaborative Innovation Center for Translational Medicine (Grant number TM201904), and Medical and Engineering Foundation of Shanghai Jiao Tong University (Grant numberWF540162605).

## Conflict of Interest

The authors declare that the research was conducted in the absence of any commercial or financial relationships that could be construed as a potential conflict of interest.

## Publisher's Note

All claims expressed in this article are solely those of the authors and do not necessarily represent those of their affiliated organizations, or those of the publisher, the editors and the reviewers. Any product that may be evaluated in this article, or claim that may be made by its manufacturer, is not guaranteed or endorsed by the publisher.
