# Supplementary material for: Imaging Insights of Isolated Idiopathic Dystonia: Voxel-Based Morphometry and Activation Likelihood Estimation Studies
Source: Front Neurol. 2022 Apr 26;13:823882. doi: 10.3389/fneur.2022.823882 (PMC9087834; doi:10.3389/fneur.2022.823882)
Supplement: Supplementary file 1 [file Data_Sheet_1.docx]

Supplementary Material

**Supplementary Table 1. Demographic and clinical data of individuals**

| **Patient** | **Sex** | **Age**  **(Y)** | **Onset age (Y)** | **Disease duration (Year/Month)** | **Symptomatic diagnosis** | **Surgical procedures [(bilateral/left/right-stimulation targets)/ablation targets]** | **Baseline BFMDRS/TWSTRS (Total Scores)** | **Electrode** **configurations & parameters of deep brain stimulation** | **Healthy controls** | **Sex** | **Age (Y)** |
| --- | --- | --- | --- | --- | --- | --- | --- | --- | --- | --- | --- |
| 1 | Male | 33 | 32 | 1Y | Segmental dystonia (Meige syndrome) | B-GPi | 30 | R: C+0- ; 4.35V 60ms 130Hz  L: C+0-; 4.00V 60ms 130Hz | 1 | Male | 27 |
| 2 | Female | 52 | 5 | 47Y | Multifocal dystonia | B-PVP | 67 | - | 2 | Female | 52 |
| 3 | Male | 36 | 11 | 25Y | Multifocal dystonia | B-GPi | 27.5 | R: 0- 1+; 3.00V 60ms 145Hz  L: 0- 1+; 3.50V 60ms 145Hz | 3 | Male | 36 |
| 4 | Male | 45 | 44 | 1Y | Focal dystonia (jaw affected) | B-GPi | 18 | R: 1- 2+; 2.10V 50ms 135Hz  L: 1- 2+; 2.00V 50ms 135Hz | 4 | Male | 46 |
| 5 | Female | 34 | 33 | 1Y | Generalized dystonia | B-GPi | 41 | R: C+0-1-; 3.75V 60ms 160Hz  L: C+1-; 3.50V 60ms 160Hz | 5 | Female | 34 |
| 6 | Male | 10 | 9 | 1Y | Multifocal dystonia | B-GPi | 96 | R: C+0- ; 3.45V 90ms 160Hz  L: C+0-; 3.60V 90ms 160Hz | 6 | Male | 20 |
| 7 | Male | 43 | 42 | 9M | Generalized dystonia | B-GPi | 12 | R: C+0- ; 2.55V 90ms 160Hz  L: C+0-; 2.25V 90ms 160Hz | 7 | Male | 44 |
| 8 | Female | 34 | 30 | 4Y | Multifocal dystonia | B-GPi | 40 | R: C+0-1- ; 3.45V 70ms 160Hz  L: C+0-1-; 3.75V 70ms 160Hz | 8 | Female | 35 |
| 9 | Female | 72 | 71 | 5M | Segmental dystonia (jaw, neck affected) | B-GPi | 7.5 | R: C+1- ; 2.75V 70ms 145Hz  L: C+1-; 2.5V 60ms 145Hz | 9 | Female | 71 |
| 10 | Male | 72 | 66 | 6Y | Segmental dystonia (Meige syndrome) | B-GPi | 8 | R: C+0- ; 2.75V 60ms 145Hz  L: C+0-; 2.95V 60ms 170Hz | 10 | Male | 71 |
| 11 | Female | 50 | 35 | 15Y | Segmental dystonia (Meige syndrome) | B-STN | 11 | R: C+2- ; 2.00V 60ms 135Hz  L: C+2-; 2.25V 60ms 135Hz | 11 | Female | 29 |
| 12 | Male | 68 | 65 | 3Y | Segmental dystonia (Meige syndrome) | B-GPi | 10 | R: C+1- ; 2.75V 60ms 140Hz  L: C+0-; 2.75V 60ms 140Hz | 12 | Male | 71 |
| 13 | Male | 45 | 39 | 6Y | Multifocal dystonia | B-GPi | 49 | R: C+0- ; 3.00V 70ms 160Hz  L: C+0-1-; 2.35V 60ms 160Hz | 13 | Male | 51 |
| 14 | Male | 16 | 15 | 9M | Generalized dystonia | B-GPi | 12 | R: C+0- ; 3.00V 90ms 170Hz  L: C+0-; 3.00V 90ms 170Hz | 14 | Male | 21 |
| 15 | Female | 25 | 17 | 8Y | Multifocal dystonia | B-GPi | 35 | R: C+0- ; 3.20V 90ms 160Hz  L: C+0-; 3.50V 90ms 160Hz | 15 | Female | 25 |
| 16 | Male | 6 | 5 | 1Y | Multifocal dystonia | B-GPi | 82 | R: C+0- ; 3.75V 90ms 160Hz  L: C+0-; 4.05V 90ms 160Hz | 16 | Male | 20 |
| 17 | Female | 55 | 54 | 1Y | Segmental dystonia (Meige syndrome) | B-GPi | 18.5 | R: 0-1+ ; 2.75V 70ms 130Hz  L: 0-1+; 2.95V 70ms 130Hz | 17 | Female | 57 |
| 18 | Female | 30 | 20 | 10Y | Focal dystonia (Cervical dystonia) | B-GPi | 45 | R: C+0- ; 3.10V 90ms 160Hz  L: C+0-; 3.45V 90ms 160Hz | 18 | Female | 28 |
| 19 | Female | 48 | 47 | 8M | Segmental dystonia (Meige syndrome) | B-GPi | 11 | R: 0+1- ; 2.5V 60ms 145Hz  L: 0+1-; 2.50V 60ms 145Hz | 19 | Female | 51 |
| 20 | Male | 40 | 18 | 22Y | Generalized dystonia | B-GPi | 66.5 | R: C+0- ; 3.75V 90ms 160Hz  L: C+0-; 4.00V 90ms 160Hz | 20 | Male | 36 |
| 21 | Female | 63 | 57 | 6Y | Generalized dystonia | B-GPi | 19 | R: C+0- ; 3.20V 60ms 160Hz  L: C+0-; 3.20V 60ms 160Hz | 21 | Female | 63 |
| 22 | Female | 28 | 7 | 21Y | Generalized dystonia | B-GPi | 69 | R: C+0- ; 3.45V 60ms 140Hz  L: C+0-; 3.25V 60ms 140Hz | 22 | Female | 29 |
| 23 | Female | 54 | 46 | 8Y | Focal dystonia (Cervical dystonia) | B-GPi | 40.5 | R: C+0-1- ; 3.00V 90ms 160Hz  L: C+0-1-; 3.00V 90ms 160Hz | 23 | Female | 53 |
| 24 | Male | 42 | 41 | 7M | Segmental dystonia (neck, right upper limb affected) | B-GPi | 22 | R: C+1- ; 2.85V 60ms 140Hz  L: C+1-; 3.05V 60ms 140Hz | 24 | Male | 38 |
| 25 | Female | 39 | 37 | 2Y | Segmental dystonia (jaw, neck affected) | B-GPi | 14 | R: 0+1- ; 2.75V 60ms 130Hz  L: 0+1-; 2.75V 60ms 130Hz | 25 | Female | 41 |
| 26 | Female | 16 | 11 | 5Y | Multifocal dystonia | B-GPi | 56 | R: C+0- ; 3.50V 90ms 145Hz  L: C+0-; 3.85V 90ms 145Hz | 26 | Female | 20 |
| 27 | Female | 61 | 58 | 3Y | Segmental dystonia (Meige syndrome) | B-GPi | 10.5 | R: C+0- ; 3.00V 60ms 160Hz  L: C+0-; 3.00V 60ms 160Hz | 27 | Female | 63 |
| 28 | Female | 60 | 57 | 3Y | Segmental dystonia (jaw, neck affected) | B-PVP | 26 | - | 28 | Female | 61 |
| 29 | Female | 67 | 57 | 10Y | Segmental dystonia (mouth, neck affected) | B-PVP | 18 | - | 29 | Female | 64 |
| 30 | Female | 69 | 59 | 10Y | Segmental dystonia (Meige syndrome) | B-GPi | 12 | R: C+1- ; 3.00V 70ms 160Hz  L: C+0-; 3.50V 70ms 160Hz | 30 | Female | 69 |
| 31 | Male | 71 | 66 | 5Y | Generalized dystonia | B-GPi | 35.5 | R: C+0-1-; 3.95V 90ms 160Hz  L: C+0-; 4.25V 90ms 160Hz | 31 | Male | 71 |
| 32 | Female | 60 | 56 | 4Y | Segmental dystonia (Meige syndrome) | B-GPi | 14 | R: C+0- ; 3.00V 90ms 130Hz  L: C+0-; 2.85V 90ms 130Hz | 32 | Female | 60 |
| 33 | Female | 30 | 18 | 12Y | Segmental dystonia (neck, right upper limb affected) | B-GPi | 26 | R: C+0- ; 3.20V 60ms 160Hz  L: C+0-; 3.70V 60ms 160Hz | 33 | Female | 29 |
| 34 | Male | 75 | 67 | 8Y | Segmental dystonia (Meige syndrome) | B-GPi | 11 | R: 0+1- ; 3.00V 60ms 130Hz  L:0+1-; 3.50V 60ms 130Hz | 34 | Male | 72 |
| 35 | Male | 21 | 20 | 1Y | Multifocal dystonia | B-GPi | 43.5 | R: C+0- ; 3.25V 90ms 160Hz  L: C+0-; 3.75.V 90ms 160Hz | 35 | Male | 21 |
| 36 | Female | 68 | 60 | 8Y | Segmental dystonia (Meige syndrome) | R-GPi+L-STN | 18 | R: C+0-1-; 2.50V 60ms 130Hz  L: C+2-; 2.00V 60ms 130Hz | 36 | Female | 72 |
| 37 | Female | 59 | 49 | 10Y | Segmental dystonia (Meige syndrome) | B-STN | 12 | R: C+3-; 2.25V 90ms 130Hz  L: C+2-; 2.80V 90ms 130Hz | 37 | Female | 56 |
| 38 | Male | 20 | 19 | 6M | Focal dystonia (Cervical dystonia) | L-PVP | 37 | - | 38 | Male | 22 |
| 39 | Female | 21 | 8 | 13Y | Multifocal dystonia | B-PVP | 15 | - | 39 | Female | 23 |
| 40 | Female | 44 | 42 | 2Y | Segmental dystonia (Meige syndrome) | B-STN | 11 | R: C+0- ; 3.50V 90ms 145Hz  L: C+0-; 3.00V 90ms 145Hz | 40 | Female | 45 |
| 41 | Female | 46 | 43 | 3Y | Segmental dystonia (Meige syndrome) | B-STN | 10 | R: C+0- ; 2.45V 90ms 160Hz  L: C+0-; 2.75V 90ms 160Hz | 41 | Female | 48 |
| 42 | Male | 44 | 24 | 20Y | Multifocal dystonia | B-PVP | 18 | - | 42 | Male | 48 |
| 43 | Male | 65 | 57 | 8Y | Segmental dystonia (Meige syndrome) | B-STN | 27 | R: C+0- ; 3.50V 90ms 130Hz  L: C+0-; 3.85V 90ms 130Hz | 43 | Male | 64 |
| 44 | Female | 62 | 61 | 1Y | Focal dystonia (Cervical dystonia) | R-PVP | 23 | - | 44 | Female | 62 |
| 45 | Male | 51 | 21 | 30Y | Generalized dystonia | B-PVP | 71 | - | 45 | Male | 50 |
| 46 | Female | 57 | 57 | 2M | Segmental dystonia (Meige syndrome) | B-STN | 9 | R: C+3- ; 2.35V 90ms 160Hz  L: C+2-; 2.70V 90ms 160Hz | 46 | Female | 56 |
| 47 | Male | 65 | 46 | 19Y | Focal dystonia (Cervical dystonia) | L-PVP | 18 | - | 47 | Male | 64 |
| 48 | Female | 69 | 69 | 1M | Segmental dystonia (Meige syndrome) | B-STN | 18 | R: 2+3-; 2.30V 60ms 130Hz  L: 2+3-; 2.00V 60ms 130Hz | 48 | Female | 67 |
| 49 | Female | 41 | 26 | 15Y | Generalized dystonia | B-STN | 16 | R: 2+3- ; 2.50V 90ms 140Hz  L: 2+3-; 3.45V 90ms 140Hz | 49 | Female | 46 |
| 50 | Male | 28 | 25 | 3Y | Focal dystonia (Cervical dystonia) | B-STN | 35 | R: C+3- ; 3.10V 60ms 160Hz  L: C+3-; 3.10 V 60ms 160Hz | 50 | Male | 27 |
| 51 | Male | 18 | 16 | 2Y | Multifocal dystonia | B-PVP | 40 | - | 51 | Male | 23 |
| 52 | Female | 49 | 48 | 6M | Focal dystonia (Cervical dystonia) | L-PVP | 30.5 | - | 52 | Female | 49 |
| 53 | Female | 50 | 49 | 1Y | Segmental dystonia (Meige syndrome) | B-STN | 15.5 | R: C+3- ; 2.50V 60ms 160Hz  L: C+3-; 2.75 V 60ms 160Hz | 53 | Female | 51 |
| 54 | Female | 62 | 57 | 5Y | Segmental dystonia (Meige syndrome) | B-(GPi+PVP) | 61 | R: C+0- ; 3.00V 90ms 130Hz  L: C+0-; 3.00V 90ms 130Hz | 54 | Female | 61 |
| 55 | Male | 18 | 17 | 1Y | Multifocal dystonia | B-STN | 17 | R: C+3- ; 3.50V 90ms 145Hz  L: C+3-; 3.85V 90ms 145Hz | 55 | Male | 22 |
| 56 | Male | 35 | 15 | 20Y | Generalized dystonia | B-PSA | 28 | R: C+0- ;2.70V 90ms 145Hz  L: C+0-; 2.95V 90ms 145Hz | 56 | Male | 27 |
| 57 | Male | 45 | 44 | 1Y | Focal dystonia (Cervical dystonia) | B-STN | 27.5 | R: C+0- ;2.80V 90ms 130Hz  L: C+0-; 2.60V 90ms 130Hz | 57 | Male | 46 |
| 58 | Male | 24 | 17 | 7Y | Focal dystonia (right hand affected) | L-STN | 8 | L: C+3-; 2.95V 60ms 130Hz | 58 | Male | 24 |
| 59 | Male | 19 | 17 | 2Y | Focal dystonia (left upper limb affected) | L-STN | 11 | L: C+3-; 3.25V 90ms 160Hz | 59 | Male | 23 |
| 60 | Male | 36 | 31 | 5Y | Focal dystonia (Cervical dystonia) | R-PVP | 66 | - | 60 | Male | 41 |
| 61 | Female | 50 | 49 | 1Y | Segmental dystonia (Meige syndrome) | B-GPi | 18 | R: C+1- ; 4.10V 90ms 130Hz  L: C+1-; 4.00V 90ms 130Hz | 61 | Female | 50 |
| 62 | Male | 20 | 19 | 1Y | Focal dystonia (right hand affected) | L-STN | 18 | L: C+2-; 2.55V 60ms 130Hz | 62 | Male | 22 |
| 63 | Male | 15 | 10 | 5Y | Generalized dystonia | B-STN | 22.5 | R: C+3- ; 2.50V 60ms 130Hz  L: C+3-; 2.50V 60ms 130Hz | 63 | Male | 22 |
| 64 | Male | 24 | 16 | 8Y | Multifocal dystonia | B-PVP | 31 | - | 64 | Male | 24 |
| 65 | Female | 21 | 3 | 18Y | Generalized dystonia | B-(STN+PVP) | 39 | R: C+3- ; 2.50V 90ms 145Hz  L: C+3-; 2.50V 90ms 145Hz | 65 | Female | 20 |
| 66 | Male | 50 | 46 | 4Y | Focal dystonia (Cervical dystonia) | B-(STN+PVP) | 48 | R: C+2- ; 2.95V 60ms 130Hz  L: C+3-; 3.00V 60ms 130Hz | 66 | Male | 51 |
| 67 | Male | 32 | 29 | 3Y | Focal dystonia (Cervical dystonia) | L-PVP | 49 | - | 67 | Male | 28 |
| 68 | Male | 39 | 37 | 1.5Y | Focal dystonia (Cervical dystonia) | L-PVP | 18 | - | 68 | Male | 26 |
| 69 | Female | 55 | 50 | 5Y | Segmental dystonia (Meige syndrome) | B-STN | 11 | R: C+3- ; 2.50V 60ms 130Hz  L: C+3-; 2.50V 60ms 130Hz | 69 | Female | 55 |
| 70 | Female | 60 | 55 | 5Y | Generalized dystonia | B-GPi | 40 | R: C+1- ; 4.60V 90ms 160Hz  L: C+0-1-; 4.25V 90ms 160Hz | 70 | Female | 54 |
| 71 | Male | 18 | 9 | 9Y | Segmental dystonia (mouth, neck affected) | B-GPi | 8 | R: C+1- ; 3.10V 90ms 140Hz  L: C+0-; 2.25V 90ms 140Hz | 71 | Male | 23 |
| 72 | Male | 63 | 57 | 6Y | Focal dystonia (Cervical dystonia) | B-(GPi+PVP) | 51 | R: C+0- ; 3.45V 90ms 130Hz  L: C+0-; 2.95V 90ms 130Hz | 72 | Male | 62 |
| 73 | Male | 29 | 26 | 3Y | Focal dystonia (Cervical dystonia) | B-(GPi+PVP) | 10 | R: C+0- ; 3.00V 90ms 130Hz  L: C+0-; 2.25V 90ms 130Hz | 73 | Male | 29 |

The contacts of the electrodes from the bottom to top were successively labelled as ‘0’, ‘1’, ‘2’and ‘3’. BFMDRS, Burke–Fahn–Marsden Dystonia Rating scale; TWSTRS, Toronto Western Spasmodic Torticollis Rating Scale; GPi, internal globus pallidus; STN, subthalamic nucleus; PSA, posterior subthalamic area; PVP, posteroventral pallidotomy; NA, not applicable

**Supplementary Table 2. Basic characteristics and quality scores of the literatures**

| **Study** | **First author& Reference** | **Publication year** | **Cases（Patient No.)** | **Region** | **NOS scores** |
| --- | --- | --- | --- | --- | --- |
| 1 | Ritesh A. Ramdhani ^（18）^ | 2014 | 44 | USA | 9 |
| 2 | Camila C. Piccinin ^（30）^ | 2015 | 27 | Brazil | 8 |
| 3 | Cecile Gallea ^(22)^ | 2018 | 18 | USA | 7 |
| 4 | Pavel Filip ^(24)^ | 2017 | 25 | Czech Republic | 8 |
| 5 | P. Pantano ^(25)^ | 2011 | 19 | Italy | 7 |
| 6 | Diana N. Kirke ^(26)^ | 2017 | 40 | USA | 8 |
| 7 | Cathérine C.S. Delnooz ^(27)^ | 2015 | 23 | Netherlands | 7 |
| 8 | Tino Prell ^(28)^ | 2013 | 24 | Germany | 7 |
| 9 | Antonio Cerasa^(29)^ | 2014 | 12 | Italy | 6 |
| 10 | Kristina Simonyan ^(23)^ | 2012 | 40 | USA | 8 |
| 11 | Oliver Granert ^(31)^ | 2011 | 11 | Germany | 6 |
| 12 | Tobias Mantel ^(19)^ | 2018 | 26 | Germany | 8 |
| 13 | Kirsten E. Zeuner ^(20)^ | 2015 | 22 | Germany | 6 |
| 14 | Serena Bianchi ^(21)^ | 2019 | 16 | USA | 7 |

NOS, Newcastle-Ottawa Quality Assessment Scale


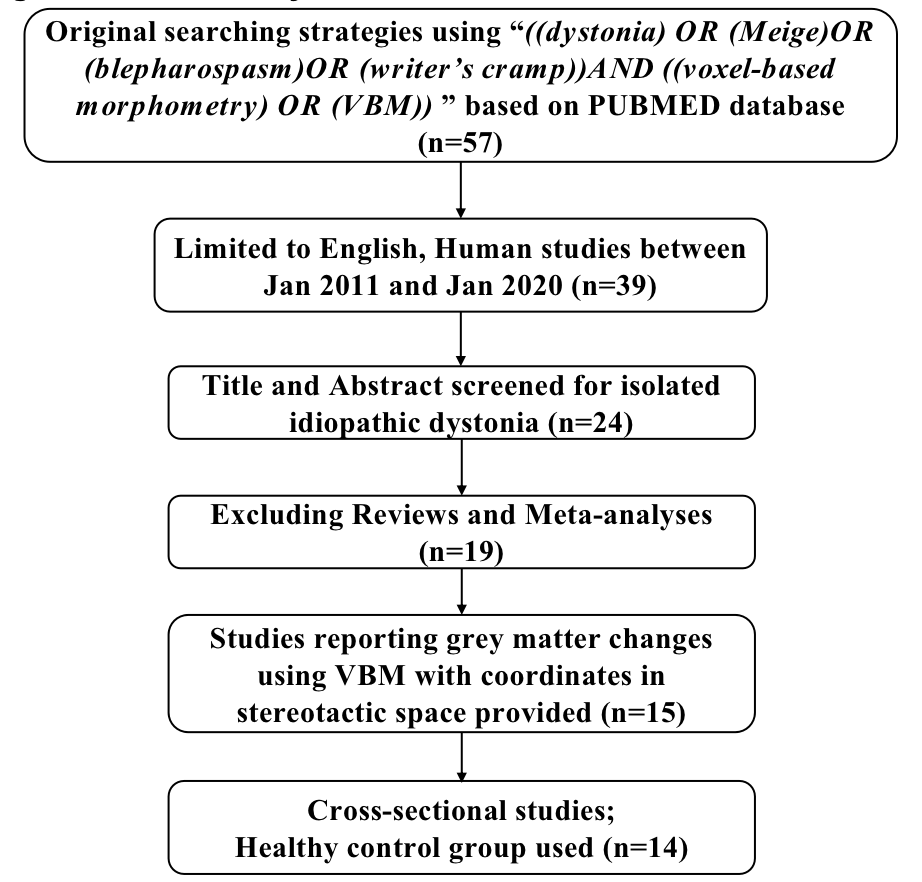
**Supplementary Figure. Searching strategies of meta-analysis**
